# Sources of Environmental Exposure to the Naturally Occurring Anabolic Steroid Ecdysterone in Horses

**DOI:** 10.3390/ani15142120

**Published:** 2025-07-17

**Authors:** Martin N. Sillence, Kathi Holt, Fang Ivy Li, Patricia A. Harris, Mitchell Coyle, Danielle M. Fitzgerald

**Affiliations:** 1 School of Biology and Environmental Science, Queensland University of Technology, Brisbane, QLD 4001, Australia; k.holtdamant@qut.edu.au (K.H.); f33.li@qut.edu.au (F.I.L.); 2 Equine Studies Group, Waltham Petcare Science Institute, Melton Mowbray LE14 4RT, UK; pat.harris@effem.com; 3 School of Agriculture and Food Sustainability, The University of Queensland, Brisbane, QLD 4072, Australia; m.coyle1@uq.edu.au (M.C.); d.smith8@uq.edu.au (D.M.F.)

**Keywords:** 20-hydroxyecdysone, NOPS (natural and other prohibited substances), muscle wasting, equine, drug testing, parasites, anabolic steroid

## Abstract

Ecdysterone controls moulting and reproduction in insects, crustaceans, and helminths. It is also produced by many plants, probably as an insect deterrent. The steroid is not made by vertebrates but has anabolic effects in mammals and could be useful for treating sarcopenia in aged horses. However, ecdysterone is banned in horseracing and equestrian sports, and with no limit of reporting, the risk of unintended exposure to this naturally occurring prohibited substance is a concern. To explore this risk, pasture plants and hay samples were analysed for ecdysterone content, as well as samples of blood, faeces, and intestinal mucosa from horses (euthanized for non-research purposes) with varying degrees of endo-parasite infestation. The variability in serum ecdysterone concentrations between different horses after administering a fixed dose was also examined. Ecdysterone was detected in 24 hay samples (0.09 to 3.74 µg/g) and several weeds, with particularly high concentrations in *Chenopodium album* (244 µg/g) and *Solanum nigrum* (233 µg/g). There was a positive correlation between faecal ecdysterone and faecal egg counts, but no effect of anthelmintic treatment and no relation to the number of encysted cyathostome larvae in the large intestine mucosa. Certain horses maintained an unusually high serum ecdysterone concentration over several weeks and/or displayed an abnormally large response to oral ecdysterone administration. Thus, the risk of environmental exposure to ecdysterone is apparent, and several factors must be considered when determining an appropriate dosage for clinical studies or setting a reporting threshold for equine sports.

## Simple Summary

Ecdysterone is a natural plant extract which is used by body builders to promote muscle strength and is undergoing clinical trials for muscle wasting in elderly people. It could also be a useful treatment for aged, retired horses, but is banned for use in competition. Currently, there is no minimum threshold for reporting the presence of ecdysterone, and positive findings for the drug have caused significant controversy. As there have been no previous studies in horses, data on the sources of environmental exposure and on the concentration of naturally acquired ecdysterone in this species are urgently needed to inform new rules of competition and to protect owners, riders, and trainers from unfair sanctions. This study explored the risk of unintended exposure through the ingestion of hay, pasture plants, and the presence of internal parasites. The results demonstrate that competition horses could be at risk of adverse analytical findings through either accidental dietary exposure or parasite infestation. Furthermore, we have discovered that some horses may be at extreme risk because their blood ecdysterone concentrations are much higher than those found in other horses, after consuming the same amount of ecdysterone. These factors need to be considered carefully when designing ecdysterone treatment regimens and when setting a reporting threshold in equine sports.

## 1. Introduction

Ecdysterone, also known as 20-hydroxyecdysone and ß-ecdysone, is a naturally occurring steroid that facilitates moulting and reproduction in insects, helminths, and crustaceans [1]. It is also found in many plants, where it is thought to have a protective effect against insect predation [2]. In fact, a comprehensive database [3] has been compiled concerning this phytoecdysteroid, which has been detected in >6% of the >2000 plant species studied [4].

Plants with high concentrations of ecdysterone have been used in traditional medicine to treat a wide range of human ailments [5], and nutritional supplements containing ecdysterone in the form of concentrated plant extracts are available commercially from many sources [4]. Although ecdysterone is not synthesised by vertebrates, it can activate oestrogen receptors (ERß) and may influence the renin–angiotensin system [6]. In fact, there are numerous experimental reports that ecdysterone can have beneficial effects on medical conditions such as diabetes, arthritis, psoriasis, atherosclerosis, liver damage, and kidney function (for a comprehensive review, see [4]). Further, ecdysteroids are being used as inducible gene switches for gene therapy [6].

Among a broad spectrum of metabolic actions, the anabolic effects of ecdysterone have received the most attention [4]. Ecdysteroids increase muscle mass in rats [6] and promote live weight gain in mice, rats, sheep, pigs, and quail [6]. Ecdysterone also increases muscle strength in young athletes [7] and is currently undergoing clinical trials as a treatment for sarcopenia in aged humans [8].

There do not appear to be any reports on the physiological effects of ecdysterone in horses. However, the potential for ecdysterone to have anabolic effects in this species presents both an opportunity and a challenge. Muscle wasting is often seen in older horses, especially those over 15 years of age [9], and so there is an opportunity to explore ecdysterone as a treatment for equine sarcopenia. At the same time, however, given its proven efficacy in human athletes and wide availability as a supplement, the challenge that faces sports drug testing authorities is one of preventing ecdysterone doping.

Advanced analytical methods to detect ecdysterone and other ecdysteroids in blood and urine samples using LC-MS/MS have been developed by sports drug testing laboratories for use in humans [10] and horses [11]. While ecdysterone has not yet been banned in human sports, the World Anti-Doping Agency has placed ecdysterone on its list of substances to be monitored [12]. Meanwhile, despite a lack of evidence for its anabolic activity in horses, and the fact that ecdysterone is a naturally occurring substance, the Federation Equestre Internationale (FEI) has taken a much harder line. The FEI has declared ecdysterone a banned anabolic substance in horses, with no lower limit of reporting, i.e., no amount of ecdysterone is permissible in a horse on the day of competition [13]. Similarly, ecdysterone would be considered a banned substance under the current Australian Rules of Racing [14], and again, no residue limit has been set. In fact, it would be difficult to decide on such a limit in the absence of relevant data gathered through studies in horses.

In 2022, two cases of adverse analytical findings for ecdysterone in competition horses were reported to harness racing and equestrian authorities by the Australian Racing Forensic Laboratories [15]. While such findings constitute a breach of the rules, the implication that ecdysterone doping had taken place was contestable. In research studies, ecdysterone of dietary origin has been detected in human urine samples at concentrations between 1 ng/mL (the detection limit of the assay) and over 2000 ng/mL [16], as well as in blood and urine samples from dogs, monkeys, sheep, cattle, rabbits, mice, and rats [17], plus insectivorous birds [18] and bats [19], at concentrations ranging from 0.1 ng/mL to 3043 ng/mL. Furthermore, there is evidence that high serum concentrations of ecdysterone are associated with parasite infestations in humans [20], and during the 1980s, there was significant interest in using the hormone as a clinical marker for the presence of internal parasites [20,21]. Thus, even with a highly specific and sensitive ecdysterone assay in place, sports authorities are still faced with the challenge of differentiating between unintentional exposure to the compound and the deliberate administration of ecdysterone supplements.

Several aims were achieved in the present study. The first aim was to determine the likelihood of environmental exposure to ecdysterone in horses by surveying a large horse breeding property for the presence of plants and/or feed ingredients with a high ecdysterone content (Study 1). Our second aim was to determine the prevalence of ecdysterone in Australian hay by examining 24 samples collected from four states, over a geographic area spanning more than 2000 km (Study 2). Our third aim was to study the relationship between ecdysterone and the presence of internal parasites, both in the intestinal lumen (Study 3) and in larvae encysted in the intestinal wall (Study 4). The potential use of serum or faecal ecdysterone concentrations as a marker for parasite infestation was also considered.

Finally, studies in humans and other mammalian species have shown that only ca. 1% of ecdysterone is measurable in the blood following oral administration [4,5]. Therefore, a small difference in the ability to absorb or metabolise the steroid could lead to a relatively large difference between individuals in the blood concentration of ecdysterone reached after consuming a certain plant or feedstuff. Understanding the extent of this variability would be important when determining dosage regimens or setting a reporting threshold for horses. Therefore, our last aim was to measure the range of serum ecdysterone concentrations attained in a group of horses given a fixed dose and sampled after the same time interval (Study 5).

## 2. Materials and Methods

### 2.1. Study 1: Ecdysterone in Pasture Plants, Feed, and Feed Supplements

In August 2023 (winter season, southern hemisphere), 50 plant specimens (Table A1) were collected over a 2-day period from a 500 Ha horse breeding property in the Riverina Murray region of New South Wales (NSW), Australia. Each plant was photographed in situ at the time of collection, later the same day once the specimen was mounted for pressing, and again five days later when the specimen had been transported to Queensland University of Technology (QUT; Brisbane, Australia) for ecdysterone analysis. Plant identity was verified at each stage using plant recognition software (PictureThis, Version 5.21.0, Glority Global, WanChai, Hong Kong). The identity of any specimens found to contain high concentrations of ecdysterone was also confirmed by a plant/weed expert at Queensland University of Technology (Professor J. Firn), and voucher specimens were lodged with the NSW herbarium for final confirmation. Two samples of hay (Teff and Lucerne/Alfalfa), seven liquid feed supplements, and three solid feed supplements were also collected from the horse property and analysed for ecdysterone.

### 2.2. Study 2: Ecdysterone in Samples of Australian Hay

**Figure 1:**
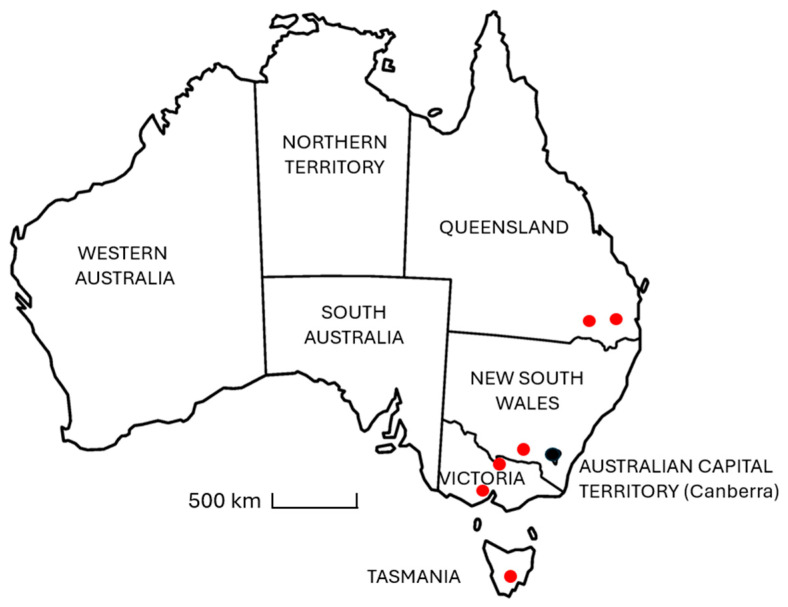
Approximate location (red dots) of hay samples collected in eastern Australia and analysed for ecdysterone content.

In December 2024, 24 samples of hay (Table A2) of different varieties were collected from horse properties and equine feedstock suppliers located in each of the following states, Queensland, NSW, Victoria, and Tasmania, spanning a geographic distance of approximately 2000 km along the eastern side of Australia (Figure 1). The purpose of the study was to conduct a qualitative analysis to determine if ecdysterone is commonly found in hay or not, and if so, whether concentrations are typically high, medium, or low relative to other plant species, without restricting the study to a particular variety, cultivar, or geographic region.

All samples were collected as a single grab sample of ca. 100 g from the surface of square bales. The samples were placed into airtight bags and stored for up to 5 days at ambient temperature (25 to 30 °C) during transport to the laboratory at Queensland University of Technology. On arrival at the laboratory, the samples were dried before ecdysterone was extracted and assayed, as described below in Section 2.6 and Section 2.7, respectively.

### 2.3. Study 3: Ecdysterone, Internal Parasite Infestation, and the Effect of De-Worming

Study 3 was conducted in accordance with the Australian Code for the Care and Use of Animals for Scientific Purposes and with approval from the Animal Ethics Committees of The University of Queensland (UQ: 2024/AE000064) and Queensland University of Technology (QUT: 2024/8515).

One week before the main study, screening was conducted on 29 Standardbred horses belonging to the teaching herd at The University of Queensland (UQ). The horses were being maintained on pasture and had received regular treatment with a broad-spectrum anthelmintic (ivermectin plus praziquantel) but had not been treated in the 3 months leading up to the study. On the day of screening, the horses were led to individual yards at the UQ Horse Unit from two paddocks—a mare paddock and a gelding paddock. A blood sample (10 mL) was taken from each horse by jugular venipuncture, and the horses were monitored closely. A fresh sample of faeces comprising three pellets was collected from the ground as soon as each horse defecated. Each sample was placed into an airtight plastic bag, with an additional sample (ca. 5 g) placed into a 20 mL sample storage bottle with an airtight lid. The samples were labelled and refrigerated immediately to reduce microbial contamination and the degradation of any parasite eggs. The horses were then returned to their paddocks.

The bagged faecal samples were transported the same day to a local testing service (EggScope, Kholo, QLD, Australia) where they were analysed using a modified McMaster method [22] for the presence of nematode eggs (strongyles), tapeworms, and coccidia. This assay has a sensitivity limit of 25 eggs/g of faeces. It has limited accuracy due to the variable nature of egg elimination and the uneven distribution of eggs within the faeces. Nevertheless, this measurement can be used to broadly classify horses as low shedders (<200 eggs/g), medium shedders (200 to 499 eggs/g), or high shedders (500+ eggs/g).

The 5 g bottled faecal samples were transported on the day of collection to the laboratory at Queensland University of Technology, where they were dried overnight in an oven at 50 °C, then stored at room temperature pending ecdysterone extraction and analysis as described in Section 2.6 and Section 2.7, respectively.

Blood samples were allowed to clot by standing them at room temperature for 30 min. The samples were then centrifuged (2000× *g* for 10 min), serum was removed and frozen at −20 °C, then transported to QUT for the analysis of ecdysterone concentration.

Horses were enrolled in the main study if their faecal egg count (FEC) was >300 eggs/g or if their serum ecdysterone concentration was >0.5 ng/mL, which was 5 times higher than the sensitivity of the ecdysterone assay. The final study group included 10 mares and 10 geldings, with a mean (±SE) age of 15.1 ± 1.4 years.

#### 2.3.1. Treatment Allocation

The horses were assigned to a control or pre-treated group based on sex, FEC, and serum ecdysterone concentration, such that each group was balanced as far as possible regarding each of these variables. Two horses with low ecdysterone concentrations were included in this study because they were high shedders, and four low shedders were included because they had high ecdysterone concentrations. The final composition of each group is shown in Table 1.

#### 2.3.2. Experimental Phase

On day 1 of the study, the horses were led from their paddocks to individual yards once again. A health examination was performed, and blood and faecal samples were collected, processed, and analysed as described for the screening phase, with the additional analysis of faecal ecdysterone concentrations. The horses that had been pre-allocated to the treatment group were weighed, then given a broad-spectrum anthelmintic (Ultramax Equine, Pharmchem Australia, Eagle Farm, QLD, Australia) in the form of an oral drench containing ivermectin (10 mg/mL) and praziquantel (75 mg/mL), delivered at a dose of 1 mL/50 kg BW, according to the manufacturer’s instructions. All the mares and geldings were then returned to their respective paddocks.

Two weeks later, the horses were brought in from the paddocks and a third sample of faeces and blood was collected, as described above. The horses were then returned to the university herd.

#### 2.3.3. Data Analysis

The values for FEC and serum ecdysterone concentration spanned a wide range, with several outlying observations for individual horses. Whereas faecal ecdysterone conformed to a normal distribution, FEC did not, even after a log transformation. Therefore, the data are presented as median (range). The untransformed data for control and treated horses were compared using a non-parametric Wilcoxon signed-rank test (SigmaPlot Version15, Inpixon, Palo Alto, CA, USA).

### 2.4. Study 4: Ecdysterone and Encysted Parasites

This study was conducted with the approval of the QUT Animal Ethics Committee, which granted a tissue use certificate (AETU 2024-9101-20541) on the basis that the study did not affect the life or death of the animals in any way.

#### 2.4.1. Animals

The samples were obtained from horses euthanised at a commercial abattoir in SE Queensland for non-research purposes. Twelve digestive tracts were obtained at random from a large herd of horses comprising a mixture of breeds (mostly Thoroughbred and Standardbred), ages, and sexes. The tissue samples could not be associated with their individual donors due to operating conditions at the abattoir.

#### 2.4.2. Sampling and Processing Procedures

Faecal samples were collected from the rectum post-mortem, and the caecum was removed from each digestive tract. This part of the digestive system was chosen based on an earlier study (unpublished), which measured total worm counts throughout the digestive tract, including adult luminal parasites and encysted larvae in the caecum, dorsal colon, and ventral colon.

The caeca were inverted, washed under a steady stream of cold tap water, blotted dry, and weighed. A section of tissue weighing not less than 10% of the total caecum weight was removed and placed in a plastic bag on ice for transport to the laboratory.

The internal layer of intestinal mucosa was scraped from each tissue sample and the external serosa layer was discarded. Small portions of mucosa (ca. 1 g) were stored at −20 °C in sealed containers, until they were analysed for ecdysterone concentration. The remaining mucosa was weighed, then placed in a flask containing warm water, pepsin, and concentrated hydrochloric acid.

The flasks were incubated at 45 °C for up to 4 h until the mucosal tissue was digested, and no large particles remained. A measured amount of formalin was added to each digest to preserve the larvae until they were counted.

The samples were filtered through a fine mesh to remove any tissue debris, transferred to a gridded Petri-dish, and examined by a trained observer using a stereo microscope. The larvae were classified according to their size, as early L3 (EL3; 0.5 to 1 mm), developing L3 (DL3; >1 mm to 3 mm), or late L3 and mucosal L4 larvae (LL3/L4; >3 mm).

Faecal samples were dried overnight at 50 °C. The dried faecal samples and frozen mucosal tissue were placed in assay buffer and pulverised using a Tissue Lyser (Qiagen, Hilden, Germany). The samples were then diluted as required and analysed using an ELISA (Invitrogen; Thermo Fisher, Scoresby, Australia).

#### 2.4.3. Data Analysis

The data were assessed for a normal distribution using a Shapiro–Wilk test, and associations between the different variables were sought using linear regression analysis. The results are presented as mean ± SE.

### 2.5. Study 5: Individual Variation in Response to a Fixed Dose of Ecdysterone

Study 5 was conducted in accordance with the Australian Code for the Care and Use of Animals for Scientific Purposes and with approval from the QUT Animal Ethics Committee (AE 2025-9579-23598).

Seventeen horses at a commercial horse breeding property in NSW, Australia, were brought in from a paddock, weighed using electronic scales, then placed in individual stalls in a covered barn. The horses were not fasted and had free access to Teff hay and water throughout the study. A sample of the hay was analysed and was found to contain ecdysterone at a concentration of 3.62 µg/g dry weight. The horses remained in the stables for 24 h before any blood samples were taken to allow any ecdysterone that might have been consumed via pasture plants to be excreted. On the day of the study, a blood sample (10 mL) was collected from each horse by jugular venipuncture and processed to obtain serum for the analysis of baseline ecdysterone concentrations, as described in Section 2.3.

Each horse was then treated with ecdysterone (Steraloids, Newport, RI, USA) at a dose of 5 mg/kg BW, suspended at a concentration of 50 mg/mL in apple sauce, and delivered orally using a syringe. The horses were treated at 5 min intervals to allow a second (10 mL) blood sample to be taken from each horse after 40 min, which is the average time for ecdysterone concentrations to peak, based on a preliminary (unpublished) pharmacokinetic study in horses. The post-administration change in ecdysterone concentration was calculated for each horse.

#### Data Analysis

The data were assessed for a normal distribution using a Shapiro–Wilk test. Pre- and post-treatment ecdysterone concentrations were compared in subgroups of horses using a paired Student’s *T*-test or a Wilcoxon signed-rank test, as appropriate. The results are presented as mean ± SE and as median (range).

### 2.6. Ecdysterone Extraction

Ecdysterone was extracted from the plant and feed samples using a modification of two published methods [23,24]. Each sample was dried overnight in an oven at 50 °C. A portion of the dried sample (20 to 30 mg) was weighed, placed in a 2 mL Eppendorf tube, then pulverised for 3 min at 30 Hz using a Tissue Lyser (Qiagen, Hilden, Germany). A solvent mixture (2 mL) comprising methanol, ethanol, and purified water in the ratio of 25:20:45 *v/v/v* was then added to the powdered sample. The sample and solvent mixture were sonicated for 30 min, then heated for 2 h at 75 °C using a heating block (Corning, Mulgrave, VIC, Australia). The sample was then allowed to cool before being centrifuged (30 s at 21,300× *g*) to pellet the solid material. The solvent was transferred to another 2 mL Eppendorf tube, and the extraction process was repeated twice more. The three portions of solvent extract were combined, then the solvent was evaporated using a centrifuge concentrator (Concentrator Plus, Eppendorf, South Pacific, Northern Sydney, NSW, Australia). The dried samples were stored overnight at 4 °C, then reconstituted using an aqueous assay buffer supplied with the assay kit, before being analysed for immunoreactive ecdysterone concentration.

For dried faeces, a sub-sample (25 to 30 mg) was placed in a 2 mL Eppendorf tube. Assay buffer was added (2 mL), and the mixture was vortexed for 1 min, sonicated for 30 min, then centrifuged for 30 s (21,300× *g*). The supernatant was then removed and assayed.

Ecdysterone concentrations in liquid feed supplements and in blood serum were much lower than those found in plants and faecal samples. These samples were measured without extraction, as preliminary results showed that the dried solvent mixture caused significant interference at the low end of the assay range. Serum samples with high concentrations of immunoreactive ecdysterone were diluted in assay buffer if necessary.

### 2.7. Ecdysterone Assay

Concentrations of immunoreactive ecdysterone were measured using a competitive ELISA kit originally developed for use with tissue extracts from haemolymph, plants, and arthropods (Invitrogen; ThermoFisher, Scoresby, VIC, Australia). According to the manufacturer, the ELISA kit has <1% cross-reactivity with mammalian steroids such as ß-oestradiol, testosterone, cortisol, corticosterone, and 7-dehydrocholesterol. In contrast to other ecdysteroid immunoassays, this kit also has low cross-reactivity with other ecdysteroids, including makisterone (5.9%), ecdysone (0.71%), and ponasterone A (0.61%). Thus, as ecdysterone is the most abundant ecdysteroid in nature [4], the assay kit may be described as having high selectivity.

Values for precision, accuracy, and recovery were determined in our laboratory for each sample matrix. For plant samples, dried spinach was used as a positive control, as this species is known to contain a relatively high concentration of ecdysterone [2]. For blood serum and faeces, blank samples that contained no detectable ecdysterone were ‘spiked’ by adding a known amount of an ecdysterone standard.

According to the manufacturer, the analytical sensitivity of the assay is 197.8 pg/mL and serum or liquid feed supplement samples above this concentration were classified as ecdysterone-positive. Typically, plants that are classified as containing ecdysterone are those that have a concentration between 0.001 and 1% (10 to 10,000 µg of ecdysterone/g of dried plant tissue). In the present study, the sensitivity threshold was set at 2 µg/g of dried material for screening pasture plants, 1.6 µg/g for dry feed samples, and 0.2 µg/g for hay samples. This corresponded to the sensitivity of the assay after diluting the sample extracts. Faecal samples were tested either without dilution or after a 1:1 dilution in assay buffer, and the limit of sensitivity was 6 ng/g of dried faeces.

To determine the intra-assay coefficient of variation, the same spinach extract, faecal sample, and serum sample were analysed six times. Accuracy and parallelism were determined using serial dilutions of spiked serum (1/1 to 1/32), spinach extract (1/500 to 1/20,000), or faecal extract (1/1 to 1/16). As accuracy also depends on the purity of the standards used, the standards supplied with the ELISA kit were compared with an analytical reference standard purchased from Sigma-Aldrich (Macquarie Park, NSW, Australia), with a European Pharmacopeia reference standard from Merck (Bayswater, VIC, Australia), and with the product administered to horses in Study 5 (Steraloids, Newport RI, USA).

Recovery was assessed by repeated extractions of a plant sample until no more ecdysterone was detectable, and by spiking a serum or faecal extract that contained no detectable ecdysterone with a known amount of ecdysterone standard, then mixing, centrifuging, evaporating, reconstituting, and assaying the sample as normal.

## 3. Results and Discussion

### 3.1. Study 1: Ecdysterone in Pasture Plants, Feed, and Feed Supplements

Despite relatively dry conditions and the recent spraying of weeds, 50 different plant species were found on the property in NSW (Table A1). Most of these plants contained < 2 µg of immunoreactive ecdysterone/g. Higher concentrations of ecdysterone were detected in eight species, however, including two species with very high concentrations in the range found in a positive control sample of spinach (Table 2).

In earlier work, ecdysterone was detected in the seeds of ca. 6% of plant species studied [4]. Thus, it was not surprising to find two plant species with high ecdysterone concentrations among the 50 species collected on the property. It should be noted, though, that this screening study was designed only to identify the presence or absence of ecdysterone in different samples, rather than to produce accurate quantitative data. It is known that ecdysterone concentrations can vary considerably between different plants of the same species, and within different parts of the same plant, as the compound is stored in one part of the plant, then transported to the plant roots and leaf tips in response to insect predation [4]. No attempt was made in the present study to determine the variability within plants or between different specimens of the same species, and this should be considered a limitation of the work. The horses were not monitored to determine if they were eating the weeds, and it is known that at least one of these plants (*Solanum nigrum*) is toxic. Nevertheless, we observed that some *Chenopodium album* plants had been torn from the ground and were damaged or incomplete, consistent with disturbance by an animal. Notwithstanding the study limitations, overall, our results do confirm that environmental exposure of horses to ecdysterone via the ingestion of specific plants is a credible risk for horse owners and a possible challenge for regulatory authorities.

None of the dry feed or dry feed-supplement samples contained immunoreactive ecdysterone at >1.6 µg/g. Three liquid feed samples contained ecdysterone at 0.05 µg/mL, 0.11 µg/mL, and 0.39 µg/mL. The supplement with the highest ecdysterone content is marketed as a herbal alternative to the analgesic, anti-inflammatory drug phenylbutazone and contains a variety of ingredients, including burdock extract, devil’s claw, elecampane, guaiacum extract, maritime pine bark, raw apple cider vinegar, rosehips, white willow, bark, yarrow, Dr Bach^TM^ flowers, honeysuckle, rescue remedy, vine, and walnut [25]. The recommended dose is 40 mL/day, equivalent to 15.6 µg/ecdysterone/day, or 0.03 µg/kg BW. To put this in context, this is ~5000 times lower than the lowest dose (0.15 mg/kg BW) reported to have an anabolic effect in humans [7]. Thus, the feed ingredients from this property were not found to present a significant risk of unintended exposure to this prohibited steroid. Nevertheless, many plant-derived supplements are available commercially, and we cannot rule out the possibility that some of these may contain higher concentrations of ecdysterone than those seen in the present study.

### 3.2. Study 2: Ecdysterone in Samples of Australian Hay

Ecdysterone was detected in all 24 samples of Australian hay (Table 3). One sample of Lucerne hay collected in Victoria (sample #8) was found to have a comparatively high concentration (14.5 µg/g) when a sub-sample was analysed for the first time. This result was confirmed when the sample extract was assayed a second time, suggesting that this was not an analytical error. To determine if the result might have been due to sample contamination, six additional sub-samples were extracted and assayed individually, revealing a much lower (mean ± SE) ecdysterone concentration of 2.71 ± 0.38 µg/g. This is consistent with the concentration range found in all other hay samples and with the likelihood that the Lucerne sample was contaminated with another plant species. Therefore, the high value was discarded, and the mean of the six replicate measurements was used in a further statistical analysis.

When the hay collections from each state were compared, there was no significant difference in mean ecdysterone concentrations between the samples collected in Victoria (1.96 ± 0.47 µg/g), NSW (1.62 ± 0.46 µg/g), and Tasmania (1.62 ± 0.53 µg/g). The samples collected in Queensland contained less ecdysterone (0.20 ± 0.03 µg/g) than those in Victoria (*p* = 0.045) but were not significantly different from those collected in the other states. The mean ecdysterone concentration of all 24 samples was 1.35 ± 0.24 µg/g, with a range (excluding the outlier) of 0.09 to 3.74 µg/g.

Overall, our results demonstrate that the presence of ecdysterone is not specific to one hay variety or one geographic location in Australia. The range of concentrations was relatively narrow and was as large within species as it was between species. As mentioned above, concentrations are likely to vary depending on several factors, including the part of the plant sampled, the degree of insect predation prior to harvest, and possibly the length of storage. Also, as the samples were collected from stockfeed suppliers, a limitation of the study is that we have no data on the growing conditions, the use of fertilisers or herbicides, etc. Thus, these data should be considered as qualitative, demonstrating that ecdysterone is a common substance in hay, that concentrations in hay are low, relative to some other plants, and that hay may contain contaminants of other plants with a higher ecdysterone content. The implication of this finding is that a nil reporting threshold for ecdysterone cannot be maintained on the basis that horse owners have a responsibility to ensure appropriate weed and parasite control measures, but rather a suitable reporting threshold needs to be set due to the presence of the hormone in this common feedstuff.

The highest concentration of ecdysterone (3.74 µg/g) was found in oaten hay (sample #11), and at an estimated consumption rate of 3% BW/day, this would deliver a daily dose of ecdysterone of ca. 0.1 mg/kg BW. The implications of this are discussed in Section 3.5, in the context of the ways in which individual horses respond to ecdysterone administration.

### 3.3. Study 3: Ecdysterone, Internal Parasite Infestation, and the Effect of De-Worming

#### 3.3.1. Association Between Faecal Egg Counts and Ecdysterone

**Figure 2:**
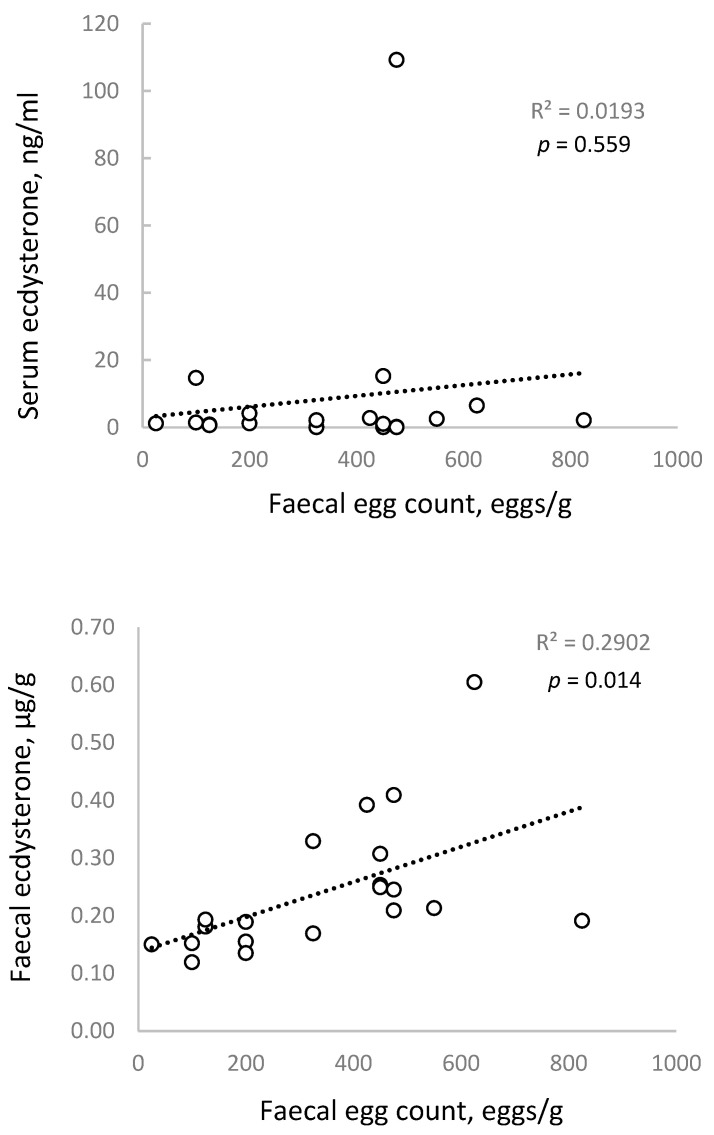
Regression analysis showing no significant association between faecal egg counts and serum ecdysterone (**top panel**) but a moderate association between faecal egg counts and faecal ecdysterone (**bottom panel**) in Standardbred horses.

A correlation analysis was performed to determine if there was a direct (linear) association between FEC and serum or faecal ecdysterone concentrations, based on the measurements obtained on day 1 of the study. Figure 2 shows a shallow trendline and no significant association between FEC and serum ecdysterone concentrations (*p* = 0.559), but a moderate and statistically significant correlation with faecal ecdysterone concentrations (*p* = 0.014).

The lack of a strong association for serum is consistent with previous studies in humans and other mammals, which have observed a similar relationship but concluded that serum ecdysterone concentrations are too variable to be a useful clinical marker for internal parasite infestations [17].

It does not appear that the association between FEC and faecal ecdysterone has been examined before. The present result is consistent with reports that internal parasites secrete ecdysterone [26] and with our previous (unpublished) observation in horses, that ecdysterone concentrations in faeces are far higher and show far less variability than those in serum.

#### 3.3.2. Effect of Anthelmintic Treatment

**Figure 3:**
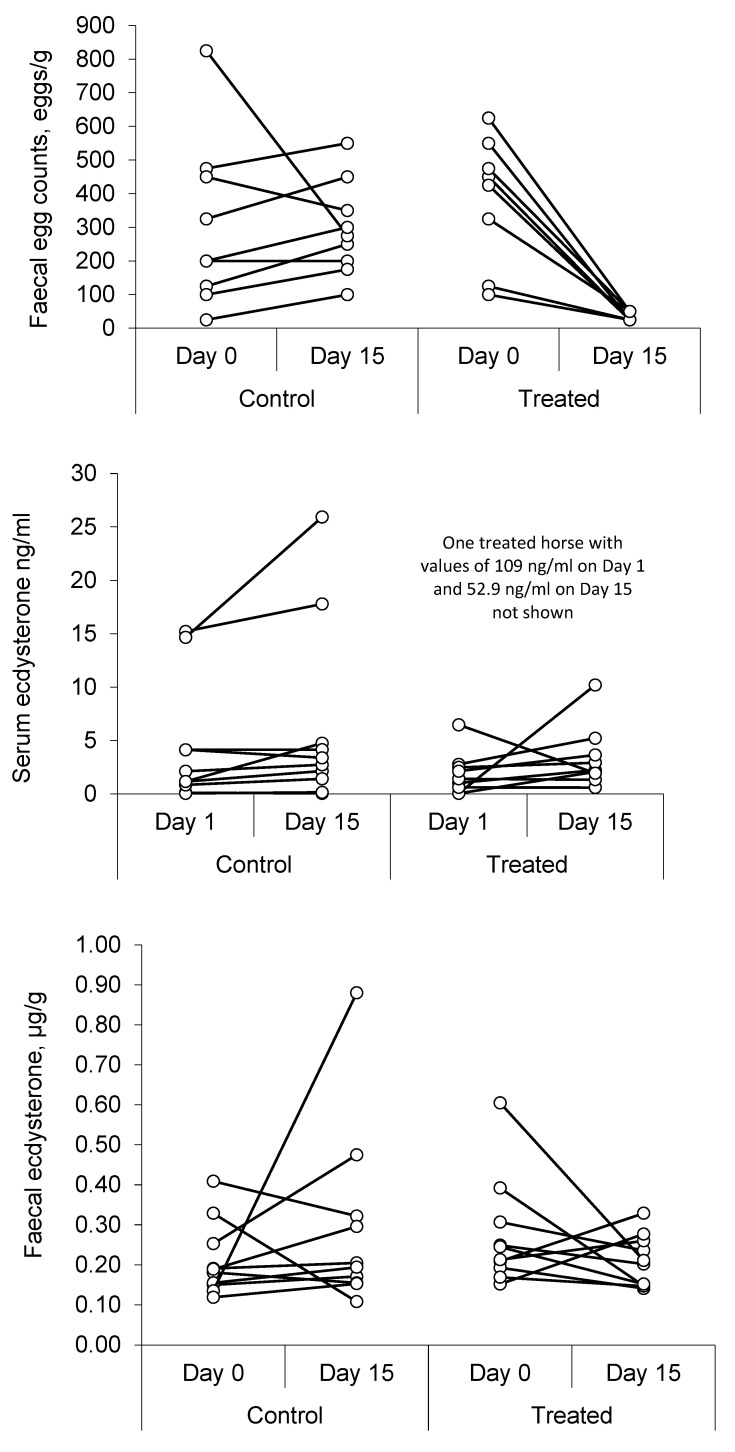
Faecal egg counts (FECs; **top panel**) and ecdysterone concentrations in serum (**middle panel**) and faeces (**bottom panel**) from individual Standardbred horses before (day 1) and after (day 15) de-worming.

Faecal analysis on day 1 showed the presence of strongyle eggs in all the samples studied, with no evidence of cestodes (tapeworms) or coccidia. Table 4 presents a summary of the results, and Figure 3 shows individual values for each horse.

The results show a marked decrease (ca. 95%) in FEC after the anthelmintic treatment (*p* = 0.002), with no significant change in the control group (*p* = 0.359) over the same period.

Serum ecdysterone concentrations varied markedly between individual horses, and one gelding returned a record value of 109 ng/mL on day 1, which was more than 60 times higher than the median value for his group. This was the same horse that returned record values during the screening phase and on day 15 after anthelmintic treatment. Overall, there was an 87% increase in the median value in control horses between day 1 and day 15 (*p* = 0.049), but no significant change in the horses that were treated (*p* = 0.557).

Faecal ecdysterone concentrations showed less variability, with no change in the median concentration between day 1 and day 15 in either group. Whereas the correlation analysis supports an association between faecal ecdysterone concentration and FEC, the results of the anthelmintic study do not. Furthermore, treatment with an anthelmintic was not effective at lowering serum ecdysterone concentrations and is unlikely, therefore, to be a suitable strategy to avoid an adverse analytical finding for ecdysterone, under the current anti-doping rules.

#### 3.3.3. Ecdysterone as a Marker for Parasite Infestation

Following the identification of ecdysterone in a wide range of insects that undergo mounting, research began to focus on other invertebrates such as snails [26] and leeches [27]. However, the studies of most practical significance to human and animal health were those in parasites, including nematodes, ascarids, and trematodes [28]. With the discovery of an association between parasites and ecdysterone in humans [17], several studies were conducted to determine if the steroid could be used as a biological marker for parasite infestation [17,20,21,28]. Early work reported an increase in serum ecdysterone in rats, hamsters, and monkeys infected with Schistosoma (liver fluke), once the parasites had developed to the egg-laying stage [21]. However, later studies reported no significant difference in serum ecdysterone between infected and uninfected individuals, in experiments using sheep, monkeys, rabbits, mice, and rats, other than an increase in rabbits, which was seen 50 days after a follow-up infection [29].

Meanwhile, a study of African patients infected with filarial parasites (Masonella perstans or Loa loa) reported that 70/100 individuals had high concentrations of ecdysterone in serum, with a mean 10 times higher than in controls, but as in the present study, these concentrations did not correlate with the concentration of present microfilaria. In fact, ecdysterone was undetectable in some heavily infected individuals [30]. Again, this phenomenon was seen in the present study, where serum ecdysterone was undetectable in two high shedders.

In a separate study, a smaller difference in serum ecdysterone was seen in humans with helminth infections [29], and the putative marker was found to be unfit for clinical use due to its limited sensitivity and specificity. Using a cut-off value of 3 nM (1443 ng/mL), it was estimated that only 34% of infected humans would show a positive result for ecdysterone, and that 5% of healthy individuals would receive a false-positive result [29].

The results of the present study confirm these much earlier findings, in that a large variation in serum ecdysterone concentrations was seen between individual horses, and that there was no significant correlation with FEC. The lack of utility of serum ecdysterone as a clinical marker of parasite infection is likely the reason that this line of research was abandoned in other species, as it has not been possible to find, within the past 30 years, any further reports of ecdysterone in mammalian blood following a parasite infestation. This could also be the reason that this potential source of environmental contamination might have escaped the notice of sports regulators.

The positive and highly probable association between faecal ecdysterone and FEC, however, does support the theory that internal parasites are a source of ecdysterone in the horse. The fact that ecdysterone concentrations did not decrease after anthelmintic treatment, however, suggests that faecal ecdysterone is not a useful marker to identify or quantify luminal parasites. This may also appear to be at odds with the association between FEC and ecdysterone seen in all horses before they were given the anthelmintic. A possible explanation for these findings is that ecdysterone is produced chiefly by parasite larvae encysted in the wall of the gut, which are not displaced by such treatments. Indeed, the production of ecdysterone during the larval development stage is more consistent with the role of ecdysterone in other developing invertebrates than in adults.

Thus, in the next study, ecdysterone concentrations in the intestinal mucosa were measured and compared with the number of encysted cyathostomes present at various life stages.

### 3.4. Study 4: Ecdysterone and Encysted Parasites

#### 3.4.1. Number of Encysted Larvae at Various Life Stages

**Figure 4:**
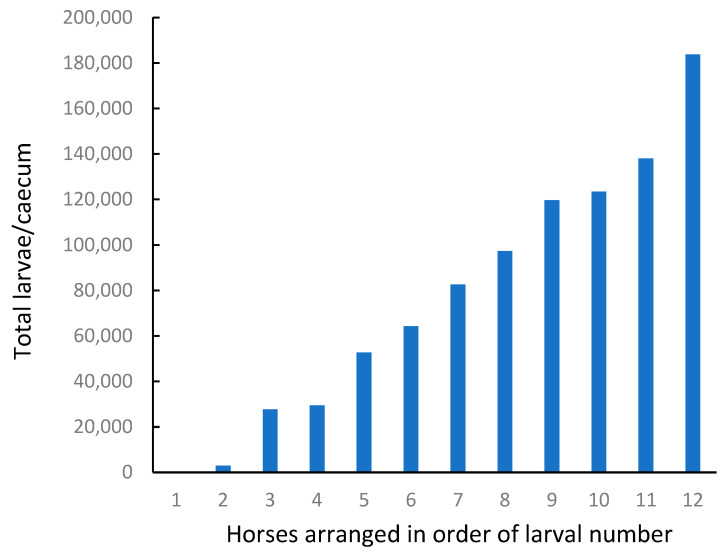
Range of values observed for the number of encysted parasite larvae in the caecal mucosa collected from 12 horses.

There was a wide range in the number of encysted larvae observed in different horses (Figure 4, Table 5), which should have been ideal for identifying an association with ecdysterone concentrations.

However, most of the larvae were classified as EL3 (early L3, 0.5 to 1 mm). There were few larvae at the developing DL3 stage, and of the twelve horses tested, nine had no parasites at the late L3 or L4 stage. Overall, the number of parasites in the LL3/L4 class was less than 1% of the total population.

These results demonstrate that encysted larvae are common and can be numerous in the intestines of some horses, while being absent in others. In the present study, based on the proportion of larvae observed at different life stages, most of the cyathostomes appeared to be dormant at the time of sampling.

Parasite larvae that are ingested at the end of the grazing season are more likely to encyst in the intestinal tract and arrest their development. They can remain in this dormant state for up to two years but typically emerge during late winter or spring (in temperate climates) when pasture conditions are favourable to the survival of parasite eggs and the larvae that hatch outside the animal. In the southern hemisphere, larvae will also remain dormant to avoid extremely hot and dry periods.

Ecdysterone is produced during the exponential growth stage of the larvae, and so based on our current observations, it could be predicted that concentrations of the hormone in the intestinal mucosa would be low. Relative to faecal samples, this proved to be the case.

#### 3.4.2. Ecdysterone Concentrations

As ecdysterone concentrations in faeces did not conform to a normal distribution, the results are presented as median [range]. Ecdysterone concentrations in the faeces (237 [200–502] ng/g) were 123 times higher than in the intestinal mucosa (1.93 [0.50–3.71] ng/g), suggesting that the faecal ecdysterone was more likely to have originated from the diet, or luminal parasites, than from encysted cyathostomes.

One horse was treated as an outlier, as the faecal ecdysterone concentration was 7893 ng/g, which is 33 times higher than the median value for the other 11 animals. The range of ecdysterone concentrations in the remaining horses was much lower in the faeces (2.5-fold) and mucosa (7.4-fold) than the range in larval numbers.

**Figure 5:**
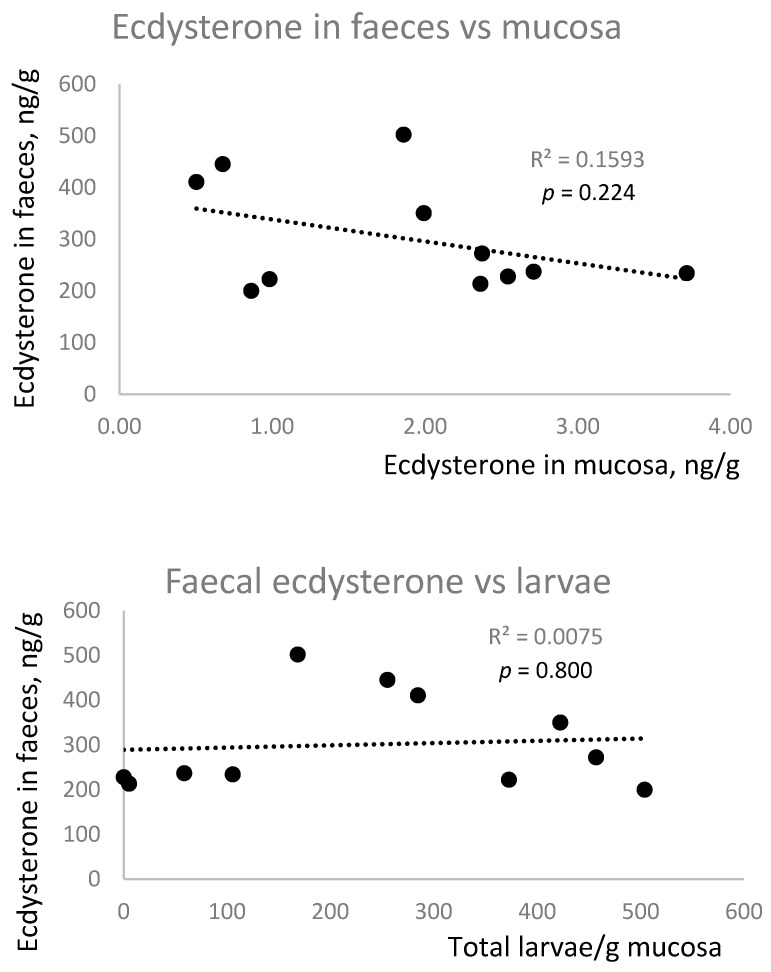

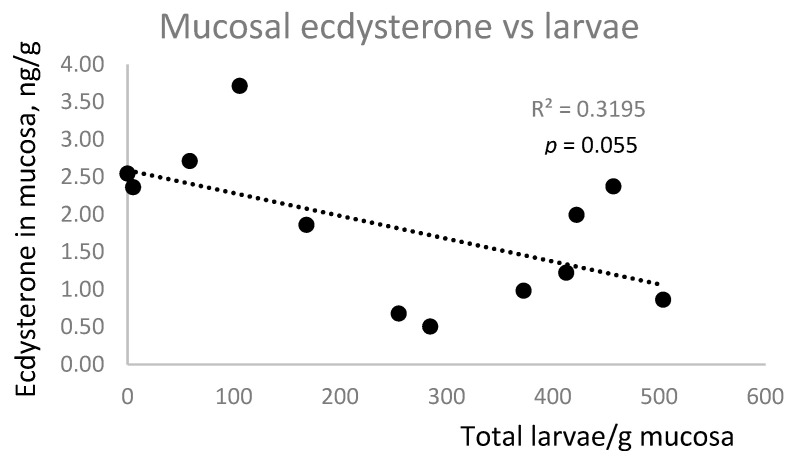
Linear regression analysis of possible associations between the concentration of ecdysterone in samples of faeces and mucosa (**top panel**) and between the density of encysted parasite larvae in the equine caecum and the ecdysterone concentration in the faeces (**middle panel**; *n* = 11) and the mucosa (**bottom panel**; *n* = 12). One horse was excluded from the analysis due to an atypically high faecal ecdysterone concentration.

There was no correlation between ecdysterone concentrations in faeces and mucosa, nor was there a positive correlation between larval number and ecdysterone concentration in faeces or mucosa (Figure 5). In fact, there was a trend (*p* = 0.055) towards a negative association between mucosal ecdysterone and larval number.

This study has demonstrated the abundance and wide range in the number of encysted parasite larvae found in the caeca of twelve horses. Unfortunately, most of the larvae in the samples in this study were classified as early L3 and were likely in a stage of growth stasis. Thus, the samples were not suitable to demonstrate a positive correlation with ecdysterone concentrations in either the mucosal tissue or the faeces, as ecdysterone is only produced during the exponential growth and development phase when the larvae transform into the L4 type and then into adult worms. To observe the proposed correlation, the study needs to be repeated during a season when pasture conditions are favourable to larval development and survival.

### 3.5. Study 5: Individual Variation in Response to a Fixed Dose of Ecdysterone

In Studies 3 and 4, horses were identified with atypically high concentrations of ecdysterone in serum and faeces, respectively. The latter observation might have resulted from the horse consuming a weed with a high ecdysterone concentration before it was euthanized and the sample collected. The former observation is more difficult to explain, however, as this horse was kept in the same paddock as nine other geldings, which had much lower ecdysterone concentrations, and a high ecdysterone concentration was observed in serum collected on three occasions, more than two weeks apart.

**Figure 6:**
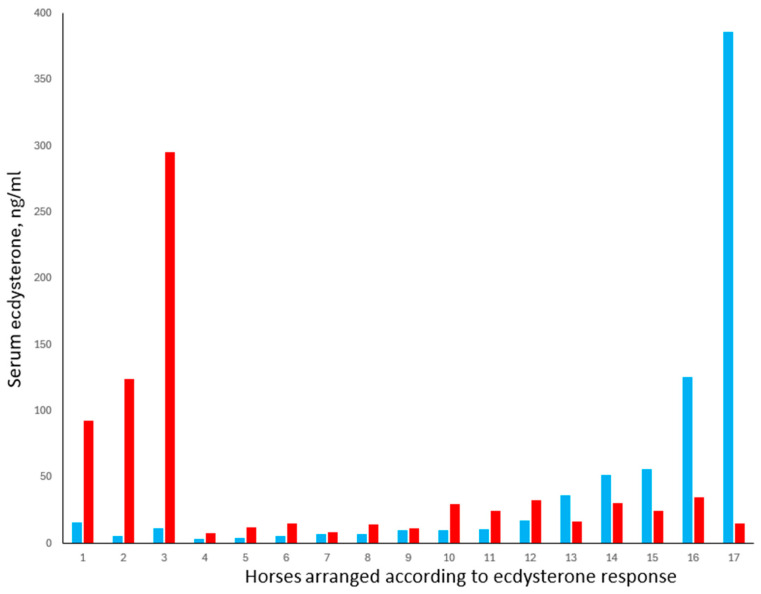
Serum ecdysterone concentrations in 17 horses sampled before (blue bars) and 40 min after (red bars) the oral administration of ecdysterone (5 mg/kg).

Study 5 was, therefore, conducted to explore the possibility that atypically high serum concentrations of ecdysterone may result in some individuals due to a difference in their ability to absorb or metabolise the compound. Serum concentrations measured in 18 horses, 40 min after ecdysterone administration, are shown in Figure 6.

The results show the complexity of predicting ecdysterone concentrations in horses and the need to consider different categories of horses. Horses 4 to 12 represented 53% of the study cohort. In this group, the mean (±SE) baseline ecdysterone concentration was 8.23 ±1.38 ng/mL, with a maximum of 16.9 ng/mL. The data conformed to a normal distribution, and following treatment, ecdysterone concentrations increased in all animals by an average of 8.77 ng/mL, i.e., approximately double the baseline concentration.

In contrast, and consistent with our hypothesis that some horses show an unusually large response to ecdysterone ingestion, three horses in the present study (17.6%) showed increases of 76.6, 118, and 283 ng/mL, which is ca. 9- to 32-fold higher than average. This is despite the fact that baseline concentrations in these horses were not different from those in horses 4 to 12. Such horses would be vulnerable, for example, to returning an adverse analytical finding if they were to compete while being exposed to plants with moderately high ecdysterone content.

The third finding from this study was that baseline ecdysterone concentrations were atypically high in at least two horses, with one animal (horse 17) having a baseline of 386 ng/mL, which is 47 times higher than the average for horses 4 to 12. This is consistent with our observation in Study 3, where one horse returned a value of 109 pg/mL within a cohort that averaged <2 ng/mL. In the present case, it appears unlikely that this was caused by the feed per se. It should be noted that all the horses received the same feed containing ca. 3.62 mg/kg of ecdysterone, and that a total daily hay intake of 3% BW would not have delivered more than 0.1 mg/kg BW of ecdysterone, assuming 100% digestibility. This is 50 times lower than the 5 mg/kg BW dose administered, which in most horses did not increase serum ecdysterone concentrations to above 30 µg/mL.

Another possibility is that the horses with a high baseline concentration of ecdysterone absorbed more ecdysterone from their feed than the others, but this too seems unlikely, as the response of these animals to ecdysterone administration was negative. Instead, the ecdysterone may have originated from internal parasites or another yet unknown source.

The last and most unexpected finding in the present study was that five horses (29%; horses 13 to 17) showed a decrease in serum ecdysterone concentrations following administration of the compound. Notably, all these horses had a baseline concentration above 36 ng/mL, which is 4.4 times higher than the average for horses 4 to 12. We have no evidence to explain the cause of this phenomenon, other than it was unrelated to breed, age, body weight, or sex. It is possible that the above-average ecdysterone concentrations were the result of endogenous ecdysterone production (e.g., by internal parasites) and that exposure to exogenous ecdysterone caused a rapid shutdown of endogenous production via a negative feedback mechanism. Alternatively, ecdysterone administration may have activated substances that interfere with the immunoassay or alter the rate of ecdysterone metabolism. These possibilities are currently being explored using LC-MS/MS analysis.

### 3.6. Ecdysterone Assay Validation

This research project entailed screening numerous samples in different matrices, with a focus on identifying different sources of environmental exposure and the variability in response between horses, rather than achieving precise quantitation or examining related ecdysteroids or their metabolites. Therefore, we chose to use a highly specific and sensitive ELISA method, rather than LC-MS/MS, which is the preferred method of drug testing laboratories. Nevertheless, the ELISA was carefully validated and proved to have good specificity, plus 10-fold greater sensitivity and an acceptable level of precision and accuracy, compared with mass spectrometry methods.

**Figure A1:**
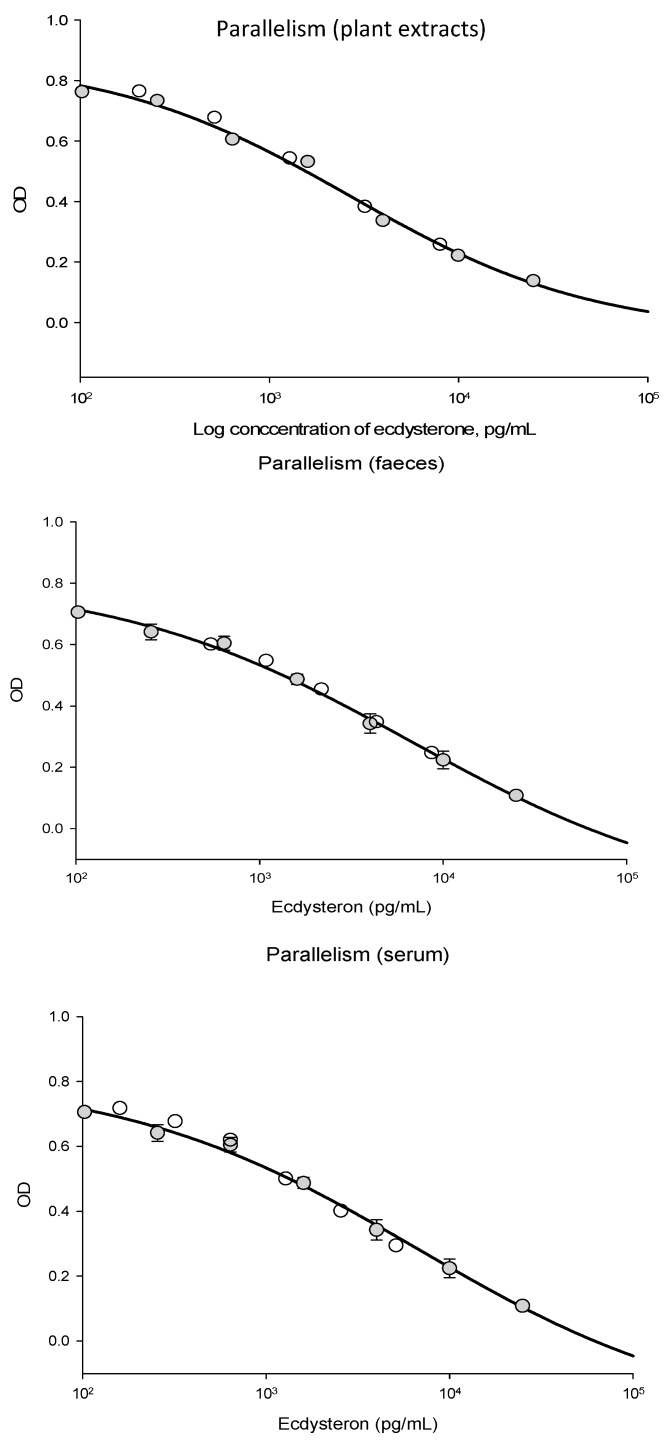
Optical density (OD) plotted against ecdysterone concentration for a set of ecdysterone standards (grey circles) and a series of dilutions of spinach extract, faeces, and serum (white circles), showing excellent parallelism for all three media.

The ecdysterone ELISA performed well when used with equine serum, faeces, and plant extracts. Parallelism between dilutions of the ecdysterone standard and dilutions of the sample was good, as shown in Figure A1. The intra-assay CV for the ecdysterone assay was 7%, which corresponded to the level of precision reported by the manufacturer. Compared to the ecdysterone standards supplied with the assay kit, the relative concentration measured was 108% for the European reference standard, 101% for the Sigma-Aldrich reference standard, and 110% for the product supplied by Steraloids. These were all considered to be within an acceptable range, given the accuracy of the assay.

Based on repeated extractions, the average (mean ± SE) recovery of ecdysterone from a sample of dried spinach was 89.6 ± 1.34% after one extraction, and 98.9 ± 0.34% after two extractions. Based on this, each plant sample in the study was extracted three times. Based on a spiked sample, the average recovery from faeces was 111 ± 4%. The average recovery from serum ranged between 115% and 104% at dilutions of 1/2 and 1/4, respectively, but was less (63%) at higher dilution rates. The immunoreactive ecdysterone concentration of a spinach sample assayed as a positive control was 211 µg/g, which is in the range of values reported previously for ecdysterone measured in spinach extracts using HPLC [31].

The ELISA was fit for the purpose of screening many samples at high sensitivity, with acceptable precision and accuracy. In cases where atypically high ecdysterone concentrations were observed, the analysis was repeated at least three times using a range of dilutions and different aliquots of serum or plant extracts. In terms of cross-reactivity with vertebrate hormones and other ecdysteroids, the selectivity of this assay is also good. However, a limitation of this study is that no information could be determined about ecdysterone metabolites, or the presence of less common ecdysteroids, of which more than 500 different analogues have been isolated and their structures identified [3]. An analysis of some of these samples by mass spectrometry could potentially yield even more information, and this is currently in progress.

## 4. Conclusions

High concentrations of ecdysterone were found in several weeds from an Australian horse breeding property. Given the difficulty in removing every weed from an extensive rural property, and the large variation in ecdysterone concentrations seen in different plants, there is a clear risk of unintentional environmental exposure to this naturally occurring prohibited substance.

Ecdysterone was detected in all samples of hay collected from a wide geographical area. The concentrations were relatively low, and a hay diet would be unlikely to deliver more than ca. 0.1 mg/kg BW per day to a horse. Nevertheless, the presence of ecdysterone in this ubiquitous feedstuff indicates that a nil reporting level for the substance is inappropriate. Furthermore, as was likely observed in the present study, hay can be contaminated with other plants that have a higher ecdysterone content. Such contamination may be hard to identify, and this presents yet another risk to horse owners.

The most striking finding from the project is the marked individual variation that occurs in ecdysterone concentrations and the observation that baseline concentrations and the response to ecdysterone ingestion can exceed a typical level by a factor of 10 to almost 50-fold in certain individuals.

In addition to environmental exposure through the ingestion of plants, the influence of internal parasites needs to be examined in future studies, as does the cause of an enhanced response to ecdysterone administration seen in some individuals, and an apparent suppression seen in others. Meanwhile, information from the present study may be useful for owners of competition horses, authorities concerned with potential ecdysterone doping in equine sports, and scientists exploring the potential clinical uses of this steroid in equine medicine.

Overall, our results show that ecdysterone is clearly present in a horse’s natural environment, that there is a likelihood of environmental exposure, and that a nil reporting threshold is inappropriate. Furthermore, we have observed that some horses would be at a greater risk of returning an adverse analytical finding than others due to their peculiar physiology that allows higher ecdysterone concentrations than normal to be reached in the blood after oral ingestion.

## Figures and Tables

**Table 1:** Allocation of horses to control and pre-treatment groups after screening for faecal egg counts (FECs) and serum ecdysterone concentrations. Values are shown as median (range).

	Control Group	Pre-Treated Group
Faecal egg count, eggs/g	338 (125–800)	363 (100–575)
Serum ecdysterone, ng/mL	1.53 (0.05 ^a^–22.1 ^b^)	1.41 (0.05 ^a^–66.5 ^c^)
Mares	4	6
Geldings	6	4
Age, years	14 (8–28)	12.5 (7–24)

^a^ one horse in each group had an ecdysterone concentration below the limit of detection, but a high FEC. For analysis, the concentration was designated as 0.05 ng/mL, which is halfway between the detection limit of the assay (0.1 ng/mL) and zero. ^b^ the highest value in this group was observed in a gelding that maintained a minimum concentration of ca. 20 ng/mL for 3 weeks during a previous pharmacokinetic study. ^c^ one gelding in the pre-treatment group returned an ecdysterone concentration of 66.5 ng/mL, which was 47 times higher than the median value and was the highest value recorded in an untreated horse to this point in time.

**Table 2:** Plants collected from an Australian horse breeding property and found to contain at least 2 µg of immunoreactive ecdysterone/g of dried sample.

Species	Common Name	Ecdysterone µg/g of Dried Plant
*Chenopodium album*	Lambsquarters	244
*Solanum nigrum*	Black nightshade	233
*Acacia pycnantha*	Golden wattle flower	2 to 5
*Rumex acetosella*	Garden sorrel	4
*Eucalyptus camaldulensis*	River red gum	3
*Cytisus proliferus*	Tree Lucerne	2
*Galium aparine*	Cleavers	2
*Acacia retinodes*	Water wattle	2

**Table 3:** Ecdysterone concentrations in 10 varieties of hay sampled throughout eastern Australia.

State	Sample #	Variety	Ecdysterone, µg/g
Queensland	4	Lucerne	0.22
	23	Teff	0.13
	1	Barley	0.09
	10	Oaten	0.18
	16	Rhodes grass hay	0.27
	2	Barley	0.31
NSW	21	Teff	3.62
	5	Lucerne	0.70
	14	Pasture	1.17
	15	Pasture	1.55
	6	Lucerne	2.07
	7	Lucerne	0.60
Victoria	8	Lucerne	2.71 (14.5) *
	11	Oaten	3.74
	12	Oaten	1.81
	9	Lucerne	0.42
	22	Teff	1.50
	17	Ryegrass	1.56
Tasmania	18	Ryegrass	1.84
	20	Sweet vernal, browntop bent	3.43
	3	Cocksfoot	0.42
	24	Wheat straw	2.83
	13	Oaten hay	0.53
	19	Ryegrass	0.66

* the value 2.71 for sample # 8 represents the average of six sub-samples. The value of 14.5 represents an outlying observation, likely due to sample contamination.

**Table 4:** Faecal egg counts and ecdysterone concentrations in Standardbred horses before and after anthelmintic treatment.

	Day 1	Day 15	*p* ^a^
Faecal egg counts, eggs/g			
Control	200 (100–825)	288 (100–550)	0.359
Treated	450 (100–550)	25 (25 ^b^–50)	0.002
Serum ecdysterone, ng/mL			
Control	1.64 (0.05 ^c^–15.2)	3.06 (0.05 ^c^–25.9)	0.049
Treated	1.77 (0.05–109.2)	2.58 (0.60–52.9)	0.557
Faecal ecdysterone, µg/g			
Control	0.19 (0.12–0.41)	0.18 (0.09–0.48)	0.846
Treated	0.23 (0.15–0.61)	0.21 (0.14–0.33)	0.322

Values are presented as median (range), *n* = 10 horses per group. ^a^ Wilcoxon signed-rank test for paired comparison between day 1 and day 15. ^b^ 25 eggs/g is the limit of sensitivity of the faecal egg assay and represents a number between 25 and 0 eggs/g. ^c^ 0.05 ng/mL is below the limit of sensitivity of the ecdysterone assay and represents a number between 0.1 and 0 ng/mL.

**Table 5:** The number of encysted cyathostome larvae at three different life stages in the caecum of 12 horses.

Stage of Development	EL3 ^a^	DL3 ^b^	LL3/L4 ^c^
Total larval population in all 12 horses	884,218	29,294	8480
Percentage of total at each life stage	95.9%	3.2%	0.9%
Median number of larvae per caecum	73,467	2804	0
Maximum number per caecum	178,068	6276	2871
Minimum number per caecum	0	0	0

^a^ early L3 (0.5 to 1 mm); ^b^ developing L3 (>1 mm to 3 mm); ^c^ late L3 and mucosal L4 larvae (>3 mm).

## Data Availability

All relevant data are contained within the manuscript.

## Abbreviations

The following abbreviations are used in this manuscript:

LC-MS/MS	Liquid chromatography–mass spectrometry/mass spectrometry
FEI	Federation Equestre Internationale
ELISA	Enzyme-linked immunosorbent assay
NSW	New South Wales
QUT	Queensland University of Technology
NHMRC	National Health and Medical Research Council
UQ	The University of Queensland
FEC	Faecal egg counts
Ca.	Approximately

## Appendix A

**Table A1:** Plants collected from an Australian horse breeding property and analysed for ecdysterone concentration.

Comnmon Name	Species	Order
Ribwort Plantain	*Plantago lanceolata*	Lamiales
Cheeseweed mallow	*Malva parviflora*	Malvales
Kurrajong	*Brachychiton populneus*	Malvales
Bitter Dock	*Rumex obtusifolius*	Caryophyllales
Common chickweed	*Stellaria media*	Caryophyllales
Garden Sorrel	*Rumex acetosella*	Caryophyllales
**Lambsquarters**	***Chenopodium album***	**Caryophyllales**
Smooth rupture wort	*Herniaria glabra*	Caryophyllales
Sticky Chickweed	*Cerastium glomeratum*	Caryophyllales
Beaked Hawk’s-beard	*Crepis vesicaria*	Asterales
Bristly Oxtongue	*Helminthotheca echioides*	Asterales
Capeweed	*Arctotheca calendula*	Asterales
Common Mugworts	*Artemisia vulgaris*	Asterales
Common Sow Thistle	*Sonchus oleraceus*	Asterales
Dandelion	*Taraxacum officinale*	Asterales
Guernsey Fleabane	*Erigion sumatrensis*	Asterales
Hairy Fleabane	*Erigeron bonariensis*	Asterales
Jersey Cudweed	*Pseudognaphalium luteoalbum*	Asterales
Purple Vipers Bugloss weed	*Echium plantagineum*	Boraginales
Rough Hawkbit	*Leontodon hispidus*	Asterales
Smooth Cats Ear	*Hypochaeris glabra*	Asterales
Spear Thistle	*Cirsium vulgare*	Asterales
Rosy Sand Crocus	*Romulea rosea*	Asparagales
Wild Garlic	*Allium ursinum*	Asparagales
Pepper tree	*Schinus molle*	Sapindales
Cleavers	*Galium aparine*	Gentianales
Burr Clover Medicks	*Medicago polymorpha*	Fabales
Golden Wattle tree	*Acacia pycnantha*	Fabales
Silver Wattle	*Acacia dealbata*	Fabales
Sub clover	*Trifolium subterraneum*	Fabales
Swedish clover	*Alsike; Trifolium*	Fabales
Tree Lucerne	*Cytisus proliferus*	Fabales
Water Wattle	*Acacia retinodes; Acacia dealbata*	Fabales
White Clover	*Triflolium repens*	Fabales
**Black nightshade**	***Solanum nigrum***	**Solanales**
Black Poplar Tree	*Populos nigra*	Malpighiales
Carnation Spurge	*Euphorbia terracina*	Malpighiales
Matted St John’s Wort	*Hypericum japonicum*	Malpighiales
St John’s Wort	*Hypericum perforatum*	Malpighiales
Gold moss Stonecrop	*Sedum*	Saxifragales
Sweetgums	*Liquidambar styraciflua*	Saxifragales
Three-part Crassula	*Crassula sieberiana*	Saxifragales
Sweet-briar rose	*Rosa rubiginosa*	Rosales
Grass Poly loose-strifes	*Lythrum hyssopifolia*	Myrtales
Red Box	*Eucalyptus polyanthemos*	Myrtales
River Red Gum	*Eucalyptus camaldulensis*	Myrtales
Reed canary grass	*Phalaris arundinacea*	Poales
Wall Barley	*Hordeum murinum*	Poales
Wild oats	*Avena fatua*	Poales
Silky Oak	*Grevillea robusta*	Proteales

The two samples indicated in bold were found to contain high concentrations of ecdysterone in the present study.

The plants collected are arranged by order, and the orders are sorted according to the frequency with which ecdysteroid-containing species are found within each order [4].

**Table A2:** Samples of hay collected from horse properties and equine stockfeed suppliers across four states in Australia and analysed for ecdysterone concentration.

State	Code	Variety	Location
Queensland	Q1	Prime lucerne	Toowoomba
	Q2	Teff	Toowoomba
	Q3	Barley	Toowoomba
	Q4	Oaten	Toowoomba
	Q5	Rhodes grass hay	Toowoomba
	Q6	Barley	Brookfield
NSW	N1	Teff	Tabletop
	N2	Lucerne	Tabletop
	N3	Pasture	Tabletop
	N4	Pasture	Lavington
	N5	Lucerne	Lavington
	N6	Lucerne	Lavington
Victoria	V1	Lucerne	Wallan
	V2	Oaten	Wallan
	V3	Oaten	Shepparton
	V4	Lucerne	Shepparton
	V5	Teff	Shepparton
	V6	Rye grass	Shepparton
Tasmania	T1	Rygrass	Hobart
	T2	Sweet vernal, browntop bent	Hobart
	T3	Cocksfoot	Hobart
	T4	Wheat straw	Hobart
	T5	Oaten hay	Hobart
	T6	Rygrass	Hobart
